# 366. Abbott BinaxNOW Rapid Antigen Test Performance in Detecting SARS-CoV-2 Infections in a COVID-19 Outbreak Among Horse Racetrack Workers

**DOI:** 10.1093/ofid/ofab466.567

**Published:** 2021-12-04

**Authors:** Krishna Surasi, Kristin J Cummings, Carl V Hanson, Mary Kate Morris, Maria Salas, David Seftel, Liza M Ortiz, Ruwan Thilakaratne, Cameron Stainken, Debra Wadford

**Affiliations:** 1 Centers for Disease Control and Prevention, Berkeley, California; 2 California Department of Public Health, Richmond, California; 3 Golden Gate Fields Medical Clinic, Berkeley, California; 4 City of Berkeley, Berkeley, California; 5 UCSF, San Francisco, California

## Abstract

**Background:**

Rapid antigen tests (e.g., Abbott’s BinaxNOW) are cheaper and faster than nucleic acid amplification tests (e.g., real-time reverse transcription polymerase chain reaction [RT-PCR]) for SARS-CoV-2 infection, with variable reported sensitivity. A horse racetrack in California experienced a COVID-19 outbreak among staff and used BinaxNOW to supplement RT-PCR. Utility of BinaxNOW in detecting SARS-CoV-2 infection in a workplace outbreak was assessed.

**Methods:**

Between November 25–December 22, 2020, anterior nasal swabs were collected from racetrack staff for six rounds of paired BinaxNOW and RT-PCR tests. BinaxNOW tests were interpreted according to manufacturer instructions. RT-PCR was performed at the state public health lab using the ThermoFisher TaqPath COVID-19 Combo Kit. Staff with positive results on either test were isolated and removed from subsequent testing. Viral cultures were attempted on specimens with cycle threshold (C_t_) < 30.

**Results:**

Overall, 769 paired results from 342 staff were analyzed. Most were of Hispanic ethnicity (62.0%) and ages ranged from 18 to 92 years (median 52). BinaxNOW performance compared to RT-PCR (95% CI) was as follows: positive percent agreement (PPA) 43.3% (34.6%–52.4%); negative percent agreement (NPA) 100% (99.4%–100%); positive predictive value (PPV) 100% (93.5%–100%); negative predictive value 89.9% (87.5%–92.0%). Among 127 RT-PCR-positive specimens, those with paired BinaxNOW-positive results (n = 55) had a lower mean C_t_ value than those with paired BinaxNOW-negative results (n = 72) (17.8 vs. 28.5) (p < 0.001). In dual positive pairs, median time from specimen collected to RT-PCR result reported was 4 days (range 1-6), compared to the 15-minute BinaxNOW reporting time. Of 100 C_t_ < 30 specimens, 51 resulted in positive virus isolation, 45 (88.2%) of which were BinaxNOW-positive.

**Conclusion:**

High NPA and PPV support immediate isolation of BinaxNOW-positive individuals, while low PPA supports confirmatory testing following BinaxNOW-negative results. BinaxNOW performed better in paired specimens with lower C_t_ value and positive viral cultures, which could suggest that among RT-PCR-positive specimens, those that are BinaxNOW-negative may be less likely to contain infectious virus than those that are BinaxNOW-positive.

**Disclosures:**

**David Seftel, M.D., M.D., M.B.A.**, **Enable Biosciences, Inc** (Board Member, Employee, Scientific Research Study Investigator, Shareholder)

